# Application of Soft Tissue Artifact Compensation Using Displacement Dependency between Anatomical Landmarks and Skin Markers

**DOI:** 10.1155/2012/123713

**Published:** 2012-07-19

**Authors:** Taebeum Ryu

**Affiliations:** Department of Industrial and Management Engineering, Hanbat National University, Daejeon 305-719, Republic of Korea

## Abstract

Soft tissue artifact is known to be one of the main sources of errors in motion analysis by means of stereophotogrammetry. Among many approaches to reduce such errors, one is to estimate the position of anatomical landmarks during a motion with joint angle or displacement of skin markers, which is the so-called compensation method of anatomical landmarks. The position of anatomical landmarks was modeled from the data of the so-called dynamic calibration, in which anatomical landmark positions are calibrated in an ad hoc motion. This study aimed to apply the compensation methods with joint angle and skin marker displacement to three lower extremity motions (walking, sit-to-stand/stand-to-sit, and step up/down) in ten healthy males and compare their reliability. To compare the methods, two sets of kinematic variables were calculated using two different marker clusters, and the difference was obtained. Results showed that the compensation method with skin marker displacement had less differences by 30–60% compared to without compensation. In addition, it had significantly less difference in some kinematic variables (7 of 18) by 25–40% compared to the compensation method with joint angle.

## 1. Introduction

Skin marker-based stereophotogrammetry is the most commonly used technique to analyze motions, despite significant errors due to the deformation of soft tissues such as skin and muscle. The displacement of skin markers relative to the underlying bones is called soft tissue artifact (STA), and it is responsible for errors in motion analysis. Skin marker displacement can be as much as 40 mm in the lower extremities [1, 2]. Error in computed angle due to STA ranges from 10° to 20° and is especially significant in abduction/adduction and internal/external rotation motions [1, 3, 4].

Methods proposed to reduce STA errors are based on either one of two principles: (1) treating the STA as an independent noise irrespective of motor tasks and (2) modeling a systematic pattern of STA in relation to motor tasks. Representatives of the first category are the studies of Challis [5], Ball and Pierrynowski [6], and Alexander and Andriacchi [7]. Challis [5] and Ball and Pierrynowski [6] made models of skin marker cluster deformation using geometric transformations, such as scaling and shearing. Alexander and Andriacchi [7] attempted to model the trajectory of skin marker displacements relative to the underlying bones using the Gaussian function.

The second category includes methods that assessed task-related patterns of STA by obtaining the positions of anatomical landmark—which indicates the skeletal pose—and skin markers at multiple postures or in an ad hoc motion. Cappello et al. [8] and Cappello et al. [9] proposed the double anatomical landmark calibration, in which anatomical landmark positions are measured by a pointer at two static postures in a motor task. Lucchetti et al. [10] proposed the so-called dynamic anatomical landmark calibration to identify anatomical landmark positions in an ad hoc motion. Instead of measuring STA skin marker displacements relative to the underlying bones, they innovatively assessed the relative movement of anatomical landmarks in reference to the coordinate frame defined by the cluster of skin markers, referred to as technical coordinate frame defined by skin markers (TCF). They modeled the displacement of anatomical landmarks against motion time or joint angle to correct anatomical landmark positions relative to TCF when performing a motor task.

As an alternative to anatomical landmark position compensation with joint angle, Ryu et al. [11] proposed anatomical landmark position compensation with skin markers. They assumed that anatomical landmark displacement is associated with skin marker displacements in the same TCF and attempted to model the relationship between them. They showed that the method was more effective than the anatomical landmark position compensation with joint angle, although they tested only by analyzing knee motions of a patient wearing an external fixator on the shank.

The present study applied the two anatomical landmark position compensation methods in real lower extremity movements of healthy people. This involved motion analysis of the hip, knee, and ankle joints in three lower extremity motions, walking, sit-to-stand/stand-to-sit, and step up/down, in 10 healthy males. The performance of the compensation method with skin markers was compared to the method with joint angle.

## 2. Methods

### 2.1. Experimental Setup

A motion measurement system with six cameras (Falcon, MotionAnalysis) was used to measure lower extremity motions (sampling frequency 60 Hz, measurement volume 4  ×  3 × 2 m). The accuracy of the system was assessed by comparing the measured distance between two marker positions to the known distance, such that the variation of the distance indicates error, as described by [12]. Mean error of the marker distance was 0.63 mm, maximum error was ±3.30 mm, and SD of the distance was 0.82 mm. 

Ten healthy young males with no previous history of musculoskeletal or neurological disorders related to the lower extremities participated in the experiment. The mean height, weight, and age of the participants were 1.75 m (SD = 0.03), 69.3 kg (SD = 5.8), and 26.2 years (SD = 3.0), respectively. All the participants signed informed consent forms. 

The anatomical coordinate frame defined by anatomical landmarks (ACF) of the pelvis, thigh, shank, and foot of the participants was defined according to [13]. Left/right anterior superior iliac spine and posterior superior iliac spine defined the ACF of the pelvis, whereas femoral head and lateral and medial epicondyles were used for the thigh. Detailed definitions of the ACFs for all lower limb segments are presented in Table 1.

**Figure 1:**
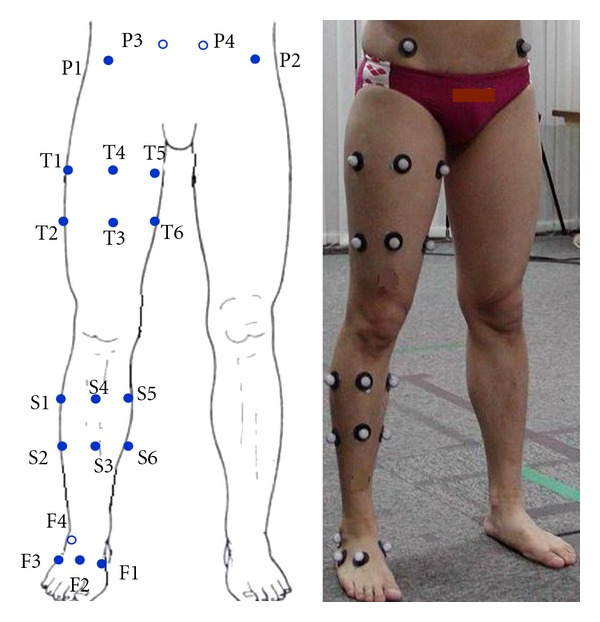
Marker placement on the participants.

Twenty reflective markers (20 mm diameter) were placed on the right lower limb segments of the participants (Figure 1). Four markers (P1–P4) are located on the palpable point of anatomical landmarks of the pelvis, and four markers (F1–F4) were placed randomly on the foot. Two sets of six markers (T1–T6 and S1–S6) were placed on the thigh and the shank, respectively. These were grouped into two marker clusters: T1–T4 and T3–T6 for the thigh and S1–S4 and S3–S6 for the shank. 

The participants performed six motor tasks: (1) standing static posture; (2) flexion/extension, abduction/adduction, and internal/external rotation of hip joint; (3) hip joint swing motion with fixed knee joint; (4) sitting static posture; (5) knee joint motion with fixed ankle; (6) walking, sit-to-stand/stand-to-sit, and step up/down. Standing and sitting static postures were held for 1-2 minutes for anatomical landmark calibration, and hip joint flexion/extension, abduction/adduction, and internal/external rotation were performed to identify the center of the hip joint. Hip joint swing motion with fixed knee and knee joint motion with fixed ankle were conducted as ad hoc motions for the dynamic anatomical landmark calibration. Walking, sit-to-stand/stand-to-sit, and step up/down were performed as target motions for analysis. 

### 2.2. Anatomical Calibration

**Figure 2:**
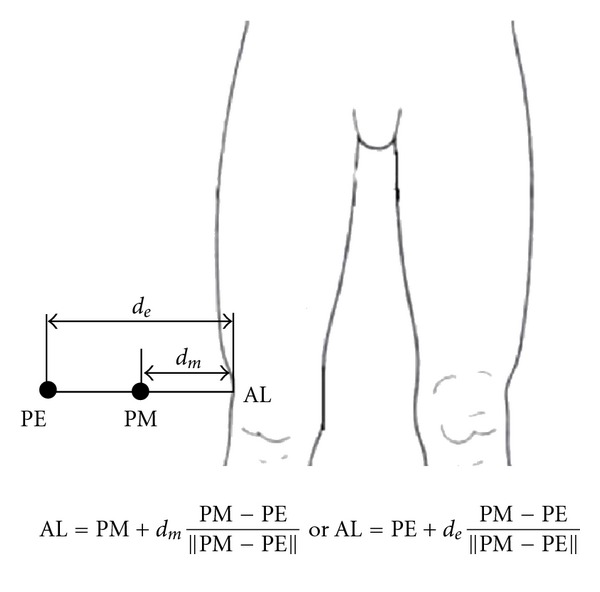
Anatomical landmark position identification with a pointer.

Anatomical landmark calibration was performed in both standing and sitting static postures using a pointer on which two markers with a known distance were mounted (see Figure 2). In the standing static posture, the position of lateral and medial epicondyles of the thigh, and that of first, second, fifth metatarsal heads, and calcaneus of the foot were identified. The position of femoral head was estimated as the center of marker trajectory in various hip motions, based on the functional method of [14]. The accuracy of the functional method was computed with the distance between two hip joint centers estimated from the pelvis and thigh. The average distance was 18 mm of [14] and 22.3 mm of this study. Then, the positions of head of fibula, tibial tuberosity, lateral, and medial malleolus of the shank were identified in the sitting posture. 

**Figure 3:**
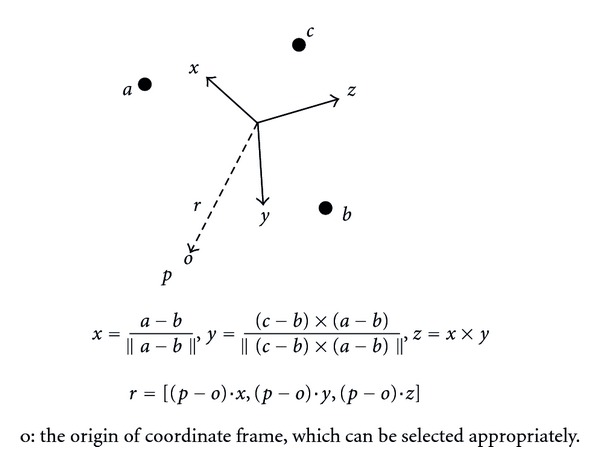
Coordinate frame definition with three markers (*a*, *b*, and *c*) and position vector of a point (*p*) relative to the frame.

**Figure 4:**
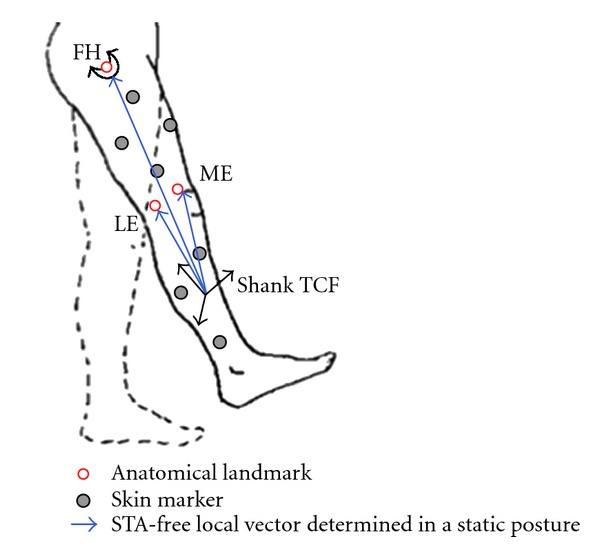
Anatomical calibration in hip joint swing motion with knee fixed (FH: femoral head, ME: medial epicondyles, LE: lateral epicondyles, TCF: technical coordinate frame).

Geometric calculations were used to determine the positions of thigh and shank anatomical landmarks relative to the TCFs on the corresponding body segments and on neighboring segments. For example, if there are three skin marker *a*, *b*, *c* and an anatomical landmark *p*, a TCF is defined by the skin markers, and the position of *p* relative to the TCF is computed like Figure 3. The anatomical landmark position relative to the TCFs on neighboring segments is computed because the neighboring segment is unaffected by STA during ad hoc motions, such as hip joint swing with knee fixed (extended) and knee joint motion with ankle fixed (dorsiflexed). Thus, this information is used to calibrate the anatomical landmark position during ad hoc motions like Figure 4. 

Two thigh TCFs (TCF^1^ by T1, T2, T3, and TCF^2^ by T4, T5, T6) and two shank TCFs (TCF^1^ by S1, S2, S3, and TCF^2^ by S4, S5, S6) were defined to compare the reliability of the two compensation methods. The difference between two values of kinematic variables, which is calculated with two sets of TCFs, was used as reliability measure. The local coordinates of each anatomical landmark of the thigh and shank were fixed in each TCF. The local coordinates of each thigh anatomical landmark were also fixed in a shank TCF and that of each shank anatomical landmark were fixed in the TCF of the foot by markers F1–F4. 

### 2.3. Pose Calculation of Coordinate Frames

The poses of all the TCFs and ACFs during ad hoc motions and three target motions were calculated using the Singular Value Decomposition algorithm of [15]. The methods find a transformation matrix that minimizes the sum of the transformation errors as in (1). The position vector and orientation matrix of each TCF was obtained from the transformation matrix, which was estimated by the algorithm between the local coordinates and global positions of the three relevant skin markers in the TCF. Likewise, the transformation matrix of each ACF was obtained from the local coordinates and estimated global positions of the relevant anatomical landmark in the ACF

(1) bi=Rai+t+ε,

where *a*_*i*_: local position vector of marker *i*, *b*_*i*_: global position vector of marker *i*, *R*: rotational matrix (3 × 3), *t*: translation vector (3 × 1), and *ε*: random error (3 × 1).

Find *R* & *t* to minimize

(2) $$\begin{array}{c} \sum_{i=1}^{n}{||Ra_{i}+t-b_{i}||}^{2}, \end{array}$$

where *n*: Number of markers (≥3).

### 2.4. Anatomical Landmark and Skin Marker Displacement

The displacements of the anatomical landmarks and skin markers on the thigh during the hip joint swing motion with fixed knee were obtained in reference to the two thigh TCFs. The positions of anatomical landmarks (lateral epicondyle [LE], medial epicondyle [ME], and femoral head [FH]) were reconstructed using a shank TCF and the relevant anatomical landmark local coordinates (3)

(3) b=Ra+t,

where *a*: local coordinate of anatomical landmark, *b*: global coordinate of anatomical landmark, *R*: rotational matrix (3 × 3) of shank TCF, and *t*: translation vector (3 × 1) of shank TCF.

Anatomical landmark displacements were calculated as the difference between the local coordinates of the reconstructed anatomical landmarks and those fixed in the standing static posture for the two thigh TCFs as in (4)

(4) $$\begin{array}{c} \text{Displacement}=l_{s}-l_{t}, \end{array}$$

where *l*_*s*_: local coordinate of anatomical landmark, which is reconstructed from shank TCF relative to thigh TCF and *l*_*t*_: local coordinate of anatomical landmarks relative thigh TCF, which is computed from static posture.

(5) ls=[(b−o)·x(b−o)·y(b−o)·z],x=si−sj||si−sj||,y=(sk−sj)×(si−sj)||(sk−sj)×(si−sj)||,z=x×y,

*b*: global coordinate of thigh anatomical landmark, *s*_*n*_: position of one marker of skin marker cluster of thigh, *n* = *i*, *j*, and *k*, and *o*: the origin of a thigh TCF, which can be selected appropriately.

Likewise, the displacement of skin markers T4 (for thigh TCF1) and T3 (for thigh TCF2) was calculated to model the anatomical landmark displacement with the displacement of a skin marker, which is not a member of a marker cluster. They were computed by subtracting the local coordinates in each thigh TCF fixed in the static posture from the measured ones during motion. In the same way, the displacements of anatomical landmarks (head of fibula, tibial tuberosity, lateral, and medial malleolus) and skin markers S4 (for shank TCF1) and S3 (for shank TCF2) during the knee joint motion with fixed ankle were obtained in reference to the two shank TCFs.

The relationship between the displacements of anatomical landmarks and skin markers was represented in a linear model using a simple regression form because of its simplicity and repeatability. Each axial component of an anatomical landmark displacement was plotted with the three axial components of the relevant skin marker displacement. The skin marker component with the highest correlation coefficient with anatomical landmark displacement was identified. It is possible to use multiple regression models with three components, but in this case, there will be multicollinearity in the model and the developed model will be much varied by analyzers. 

**Figure 5:**
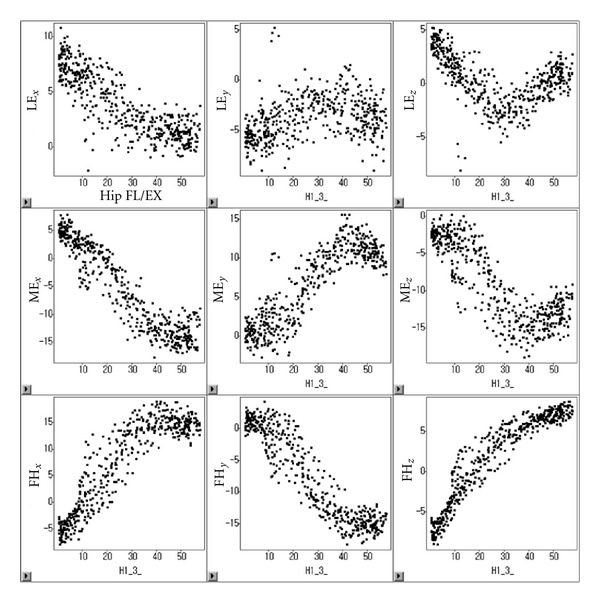
Scatter plot of anatomical landmark displacement and hip joint flexion/extension (FL/EX) of a participant (LE: lateral epicondyle, ME: medial epicondyle, FH: femoral head, *x*, *y*, and *z*: axial component of *x*, *y*, and *z*-axis).

Anatomical landmark displacement with joint rotation in the sagittal plane was modeled to compare the alternative method of [11] with the anatomical landmark compensation method with joint angle by [10]. For example, the plot between anatomical landmark displacement in the thigh and flexion/extension of hip joint was shown like Figure 5. Anatomical landmark positions during the target lower extremity motions were corrected using the developed anatomical landmark displacement models. At each frame of the motion, anatomical landmark displacements were estimated from the models. Local coordinates of the anatomical landmarks in each TCF fixed during the static posture were adjusted in relation to the relevant anatomical landmark displacements. 

### 2.5. Motion Analysis Methods

Target lower extremity motions (walking, sit-to-stand/stand-to-sit, and step up/down) were analyzed using three methods: the compensation method with skin markers [11], with joint angle [10], and Singular Value Decomposition algorithm [15]. The method of [15] was used to analyze the target motions without anatomical landmark compensation. 

### 2.6. Evaluation of Motion Analysis Reliability

Reliability of the three methods was determined by the difference between two sets of kinematic variables estimated using two marker clusters, as described by the method of [10]. The effect of STA on skin markers varies across different locations, such that the kinematic variables estimated from two different marker clusters without STA compensation will greatly differ. The kinematic variables included three angular motions (abduction/adduction, internal/external rotation, and flexion/extension) and three transitional motions (antero-posterior, longitudinal, and medio-lateral motion) of hip, knee, and ankle joint. 

### 2.7. Statistical Analysis

Two-way analysis of variance (ANOVA) was conducted to determine if motion analysis differences are affected by the type of analysis method and the type of target motion. For each kinematic variable, time series differences were obtained. Then, two-way ANOVA was performed with the type of analysis method and type of motion as independent variables. Differences found to be significantly affected by analysis method or motion type were further examined using the Student-Newman-Keuls (SNK) test to determine if the differences are statistically different from one another. 

## 3. Results 

### 3.1. Anatomical Landmark Displacement Model

**Figure 6:**
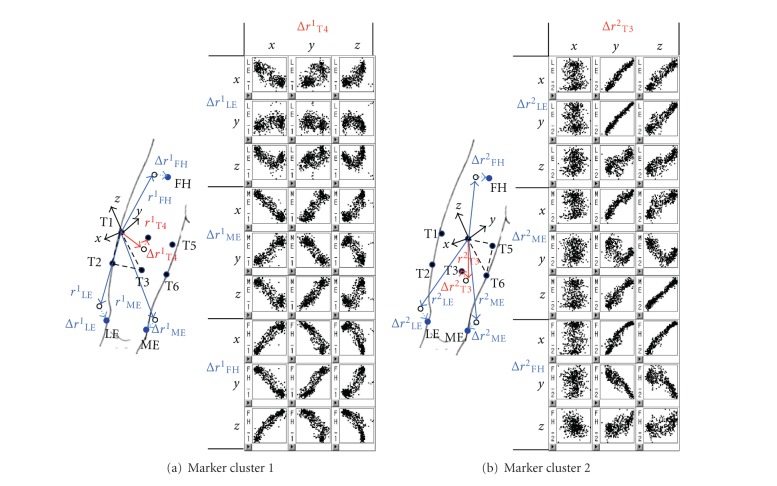
Scatter plot of anatomical landmark and skin marker displacement on the thigh of a participant (LE: lateral epicondyle, ME: medial epicondyle, FH: femoral head, *x*, *y*, and *z*: axial component of *x*, *y*, and *z*-axis).

There was a dependency between the displacements of the anatomical landmarks and the skin marker in the corresponding TCFs in the ad hoc motions. The plots between the thigh anatomical landmark displacements (Δ*r*_LE_^1^, Δ*r*_ME_^1^, Δ*r*_FH_^1^ in TCF^1^, and Δ*r*_LE_^2^, Δ*r*_ME_^2^, Δ*r*_FH_^2^ in TCF^2^) and the thigh skin marker displacements (Δ*r*_T4_^1^ in TCF^1^, Δ*r*_T3_^2^ in TCF^2^) for a participant are shown in Figure 6. Most anatomical landmark displacements had a high dependency with at least one of the three axial components of the displacements of the skin markers. However, the *y* and *z* components of Δ*r*_LE_^1^ had a weak dependency with the displacement of T4, and this is similar with the *y* component of Δ*r*_ME_^2^ and *z* component of Δ*r*_FH_^2^. Likewise, most of the shank anatomical landmark displacements (HF, TT, LM, and MM) had a high dependency with the shank skin marker displacements (S4 and S3). This study identified one axial component of the skin markers that was highly correlated with each component of anatomical landmark displacements.

A simple model for each axial component of anatomical landmark displacements was made from those having a linear regression form in relation to the axial component of the skin marker displacement having the highest correlation coefficient. The anatomical landmark displacement model was confined to linear form because it is simple to develop and it makes the anatomical displacement models consistent between model developers. For example, of a total of 42 models for anatomical landmark displacements for one participant, 32 models had R^2^ values higher than 0.5.

### 3.2. Motion Analysis Difference

**Figure 7:**
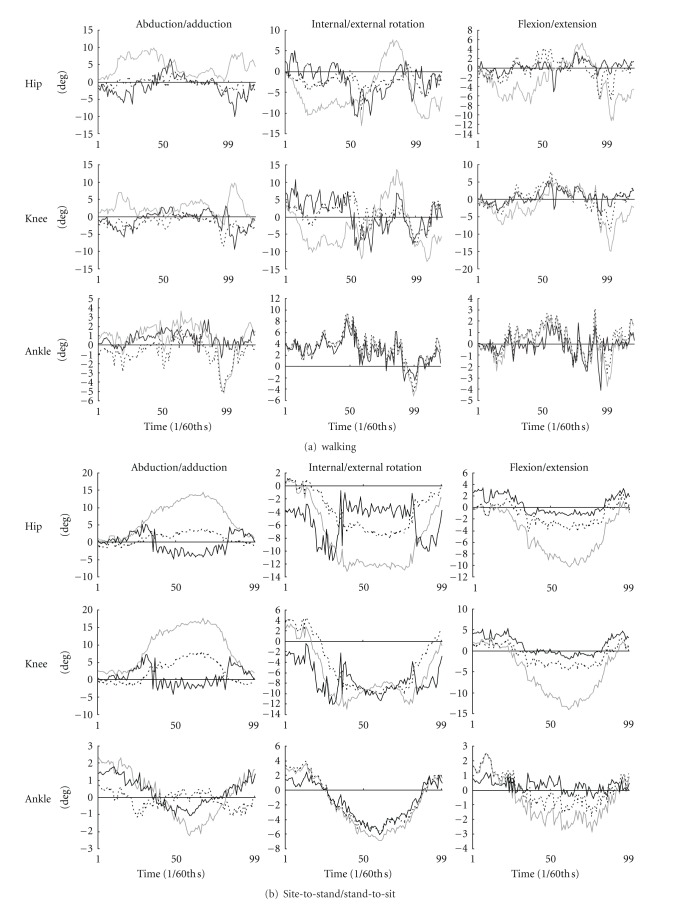
Differences between two angular motions estimated using two different marker clusters for a participant (compensation with skin marker displacement: solid black; compensation with joint angle: dashed black; without compensation: solid gray).

Figure 7 presents the differences between the joint angular motions of lower extremities estimated using two marker clusters during walking and sit-to-stand/stand-to-sit. The differences between the two estimated angular motions of the hip and knee joints are apparently more reduced with anatomical landmark compensation using skin marker displacement and joint angle than without anatomical landmark compensation. On the other hand, the differences during ankle angular motions were similar for all three methods and did not vary with compensation. These same trends were observed in step up/down motions. 

Two sets of estimated kinematic variables were calculated by averaging the differences over time for the 10 participants. Mean differences between these two sets were analyzed using two-way analysis of variance (ANOVA). The effect of motion type (walking, sit-to-stand/stand-to-sit, and step up/down), analysis method (compensation with skin marker displacement and joint angle, and without compensation), and their interaction with the mean differences for 18 kinematic variables are shown in Table 2. For most kinematic variables, the mean differences were significantly affected by the analysis method, except for the antero-posterior motion of the hip joint and the longitudinal motion of the ankle joint. They were not significantly different for varying motion types, except for internal/external rotation of all three joints. Furthermore, the interaction of the motion type and analysis method was significant only for the knee flexion/extension, ankle internal/external rotation, and hip medio-lateral motions.

**Figure 8:**
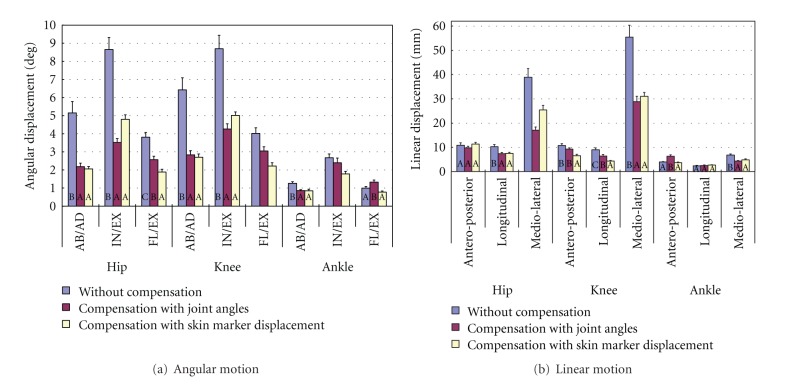
SNK test of the mean differences for different analysis methods.

**Figure 9:**
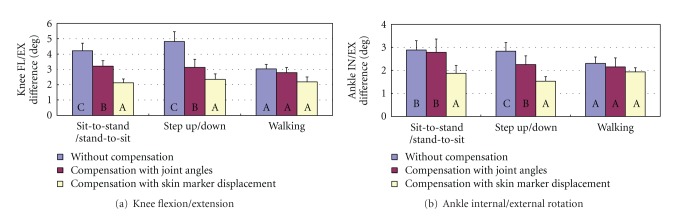
SNK test of the mean differences of knee flexion/extension and ankle internal/external rotation.

The student Newman-Keuls (SNK) test of the mean differences for the three methods showed that compensation with skin marker displacement was more effective than without compensation for most kinematic variables and more effective than compensation with joint angle for some of them. For most angular joint motions, compensation with skin marker displacement had significantly smaller (33–60%) mean differences than without compensation, except for ankle flexion/extension (see Figure 8(a)). Compensation with skin marker displacement had significantly smaller mean differences than compensation with joint angle for the flexion/extension of the hip and ankle (27 and 41%, resp.). For knee flexion/extension and ankle internal/external rotation where the interaction effect existed, compensation with skin marker displacement had significantly smaller mean differences than without compensation and compensation with joint angle in sit-to-stand/stand-to-sit and step up/down motions, but not in walking (Figure 9). 

**Figure 10:**
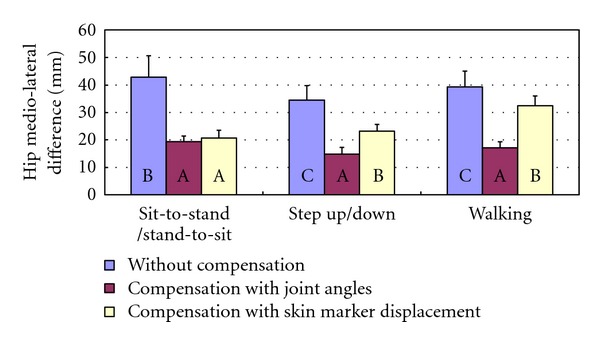
SNK test of the mean differences of hip medio-lateral motion.

Moreover, compensation with skin marker displacement had significantly smaller (27–52%) mean differences than without compensation for five of the nine linear motions (see Figure 8(b)). It also had significantly smaller (30–42%) mean differences than compensation with joint angle for three motions. For hip medio-lateral motion where the interaction effect existed, compensation with skin marker displacement had significantly smaller differences than without compensation but had significantly larger mean differences than compensation with joint angle in walking and step up/down motions (Figure 10). 

## 4. Discussion and Conclusions

Both anatomical landmark compensation methods (with skin marker displacement and joint angle) showed good reliability in real lower extremity motions. In the study, differences between two marker clusters for the hip and knee kinematic variables (all but hip antero-posterior motion) were significantly reduced by 30–60% by anatomical landmark compensation with skin marker displacement compared to that without compensation. Reduction of the differences by anatomical landmark compensation with joint angle ranged from 10 to 60%. 

Of the two anatomical landmark compensation methods, the one using skin marker displacement showed slightly better reliability in analyzing lower extremity motions. Results showed that the differences of five kinematic variables (hip flexion/extension, ankle flexion/extension, knee antero-posterior, knee longitudinal, and ankle antero-posterior motion) were significantly reduced by compensation with skin marker displacement by 30–40% more than joint angle compensation regardless of the target motion. The former method also significantly reduced the differences of knee flexion/extension and ankle internal/external rotation in sitting and stepping than the latter method by 25–30%. Compensation with joint angle was 35–50% more reliable than with skin marker displacement for only one variable (hip medio-lateral motion) in some target motions. 

Compensation with joint angle had some limitations in analyzing the kinematics of the ankle joint. While compensation with joint angle was as good as with skin marker displacement in analyzing hip and knee joint motions, it had larger mean differences than without compensation for some variables of the ankle joint. This seems to be because of the relatively large inaccuracy of the joint angle used in anatomical landmark compensation. Without compensation, the mean difference of knee flexion/extension was as large (4.0°) as that of hip flexion/extension (3.8°); the joint angle estimated without compensation was used to estimate anatomical landmark positions in compensation with joint angle. In contrast, anatomical landmark displacements of the shank (5–20 mm) were small relative to those of the thigh (15–40 mm). Therefore, the unreliable knee joint angle seems to have a large effect on the anatomical landmark position estimation of the shank relative to the thigh. 

The compensation with skin marker displacement and with joint angle still had some residual differences. Even in a stationary posture, mean differences between two sets of kinematic variables estimated using two marker clusters, which represent the instrumental errors in this study, were 0.1–3° and 1–16 mm for angular and linear motions, respectively. During target motions, mean differences for compensation with skin marker displacement and compensation with joint angle were 1–5° and 5–30 mm for angular and linear motions, respectively. 

This study had a limitation that it only evaluated the reliability of compensation methods, but not accuracy. To compute the accuracy of the compensation methods, tracking the position of underlying bones is necessary, and errors in kinematic variables should be compared to validate the compensation methods. But this study could not do it due to practical reasons, thus further study will be needed to validate the methods.

Moreover, the relationship between the displacement of the anatomical landmarks and those of skin markers reflected STA partially. STA in the thigh can occurr with hip and knee joint motions and that in the shank with knee and ankle joint motions. However, this study obtained the relationship in the thigh from only hip joint motions and those in the shank from only knee joint motions. Therefore, the relationship would not consider the whole STA of real lower extremity motions in which both the proximal and distal joints move together. A further study will be necessary to analyze and use the relationship using additional motions such as ankle joint motion with knee fixed and knee joint motion with hip fixed. 

This study applied the compensation methods in some lower extremity motions and compared their reliability. Compensation with skin marker displacement was more reliably than with joint angle, although both methods were superior to without compensation. For hip and knee motions, both anatomical landmark compensation methods reduced differences between marker clusters by half in the three motions than without compensation. Compensation with joint angle had some weaknesses in analyzing ankle motion, whereas compensation with skin marker displacement consistently showed less difference between marker clusters than without compensation.

## Figures and Tables

**Table 1:** Definition of anatomical coordinate frame (ACF) of lower extremities.

Segment	Definition
Pelvis	Origin: the mid-point of left and right anterior superior iliac spine
	*z*: connecting left anterior superior iliac spine to right anterior superior iliac spine
	*y*: orthogonal to the plane defined with left and right anterior superior iliac spine and the midpoint left and right posterior superior iliac spine
	*x*: the cross vector of *Y* and *Z*

Thigh	Origin: the midpoint of lateral and medial epicondyles
	*y*: connecting the origin to femoral head
	*x*: orthogonal to the plane defined with lateral epicondyle, medial epicondyle, and femoral head
	*z*: the cross vector of *X* and *Y*

Shank	Origin: the midpoint of lateral and medial malleolus
	*y*: intersection of the plane defined by lateral malleolus, medial malleolus, and head of fibula and the plane defined by tibial tuberosity and the midpoint of lateral and medial malleolus; positive direction is proximal
	*x*: orthogonal to the plane defined by lateral malleolus, medial malleolus, and head of fibula
	*z*: the cross vector of *X* and *Y*

Foot	Origin: calcaneus
	*y*: intersection of the plane defined by calcaneus, first metatarsal head and fifth metatarsal head, and the plane defined by calcaneus and second metatarsal head; positive direction is proximal
	*x*: orthogonal to the plane defined by calcaneus, first metatarsal head, and fifth metatarsal head
	*z*: the cross vector of *X* and *Y*

**Table 2:** ANOVA summary of mean difference of kinematic variables between marker clusters.

Joint	Kinematic variable	Motion type	Method	Motion type × Method
Hip	abduction/adduction	×	○	×
	internal/external rotation	○	○	×
	flexion/extension	×	○	×
Knee	abduction/adduction	×	○	×
	internal/external rotation	○	○	×
	flexion/extension	×	○	○
Ankle	abduction/adduction	×	○	×
	internal/external rotation	○	○	○
	flexion/extension	×	○	×

Hip	Antero-posterior (*X*)	×	×	×
	Longitudinal (*Y*)	×	○	×
	Medio-lateral (*Z*)	×	○	○
Knee	*X*	×	○	×
	*Y*	×	○	×
	*Z*	×	○	×
Ankle	*X*	×	○	×
	*Y*	×	×	×
	*Z*	×	○	×

○: significant (*α* = 0.05); ×: not significant.
